# Myoinositol as a Biomarker in Recurrent Glioblastoma Treated with Bevacizumab: A ^1^H-Magnetic Resonance Spectroscopy Study

**DOI:** 10.1371/journal.pone.0168113

**Published:** 2016-12-29

**Authors:** Eike Steidl, Ulrich Pilatus, Elke Hattingen, Joachim P. Steinbach, Friedhelm Zanella, Michael W. Ronellenfitsch, Oliver Bähr

**Affiliations:** 1 Dr. Senckenberg Institute of Neurooncology, Center for Neurology and Neurosurgery, Johann Wolfgang Goethe-University, Frankfurt/Main, Germany; 2 Institute of Neuroradiology, Center for Neurology and Neurosurgery, Johann Wolfgang Goethe-University, Frankfurt/Main, Germany; 3 German Cancer Consortium (DKTK) and German Cancer Research Center (DKFZ), Heidelberg, Germany; University of Pennsylvania, UNITED STATES

## Abstract

**Background:**

Antiangiogenic treatment of glioblastomas with Bevacizumab lacks predictive markers. Myoinositol (MI) is an organic osmolyte, with intracellular concentration changes depending on the extracellular osmolality. Since Bevacizumab markedly reduces tumor edema and influences the tumor microenvironment, we investigated whether the MI concentration in the tumor changes during therapy.

**Methods:**

We used ^1^H-magnetic resonance spectroscopy to measure the MI concentrations in the tumor and contralateral control tissue of 39 prospectively recruited patients with recurrent glioblastomas before and 8–12 weeks after starting therapy. 30 patients received Bevacizumab and 9 patients were treated with CCNU/VM26 as control. We performed a survival analysis to evaluate MI as a predictive biomarker for Bevacizumab therapy.

**Results:**

MI concentrations increased significantly during Bevacizumab therapy in tumor (p < .001) and control tissue (p = .001), but not during CCNU/VM26 treatment. For the Bevacizumab cohort, higher MI concentrations in the control tissue at baseline (p = .021) and higher differences between control and tumor tissue (delta MI, p = .011) were associated with longer survival. A Kaplan-Meier analysis showed a median OS of 164 days for patients with a deltaMI < 1,817 mmol/l and 275 days for patients with a deltaMI > 1,817 mmol/l. No differences were observed for the relative changes or the post treatment concentrations. Additionally calculated creatine concentrations showed no differences in between subgroups or between pre and post treatment measurements.

**Conclusion:**

Our data suggest that recurrent glioblastoma shows a strong metabolic reaction to Bevacizumab. Further, our results support the hypothesis that MI might be a marker for early tumor cell invasion. Pre-therapeutic MI concentrations are predictive of overall survival in patients with recurrent glioblastoma treated with Bevacizumab.

## Introduction

The use of the monoclonal VEGF blocking antibody Bevacizumab (BVZ) has a strong biological rationale in glioblastoma [1,2]. In 2009 BVZ raised attention through unprecedented response rates in recurrent glioblastoma [3,4]. In first-line therapy, the RTOG 0825 study and the AVAglio study failed to demonstrate a benefit regarding overall survival [5,6]. Recently, the results of the BELOREC trial (EORTC 26101) have been presented [7]. Bevacizumab in combination with lomustine did not result in an overall survival benefit compared to lomustine alone in glioblastoma at first recurrence. Therefore, the use of bevacizumab in first line therapy or at first recurrence is not justified. On the other hand, there are biomarkers that can identify patients that particularly benefit from bevacizumab. Unfortunately, none of these biomarkers is easily applicable and/or validated. A deeper understanding of the mechanisms of action and new biomarkers are needed to keep antiangiogenic therapy alive.

Up to now most of the research employing magnetic resonance spectroscopy (MRS) has been focused on the effects of BVZ on the tumors energy and membrane metabolism as potential markers for direct antitumoral activity [8–10]. A reversion of the increased intracellular pH, a decrease of the ratio of phosphatidylcholine to glycerophosphocholine or of the ratio of choline to N-acetyl-aspartate had been shown and interpreted as a positive therapeutic effect. Another metabolite that can be measured with ^1^H-MRS and that could be particularly relevant for antiangiogenic therapy is myoinositol (MI).

MI, which is predominantly produced by astrocytes [11], is a basic sugar and component in important molecules like inositol phosphates and phosphatidylinositol [12]. Additionally MI itself plays an important role in the cellular osmoregulation of the brain. Its concentrations seems to be variable within a wide range [13], allowing the intracellular osmolality to adapt to changes in the extracellular compartment. It has been shown in both animal and patient studies that hyponatremia for various reasons is associated with low intracellular MI levels in the brain [13–15]. The same accounts for patients with a brain edema caused by hepatic encephalopathy, even though the pathomechanism behind these disorders are fundamentally different [16–18]. In both cases the low MI concentration was reversible upon treatment of the underlying diseases. In low-grade gliomas MI is increased compared to normal appearing brain tissue. This was explained by the increased cell density of astrocytic origin in these tumors. Increased MI concentrations have also been assessed as marker for astrocytic gliosis in diseases like gliomatosis cerebri or multiple sclerosis, usually being accompanied by changes in creatine (Cre) and other metabolites [19,20]. In the context of glioblastoma MI levels in the tumor are lower than in normal appearing tissue [21–25]. This might be a consequence of the disruption of the blood-brain barrier that leads to a disturbance of the osmotic equilibrium. Interestingly, an increase of MI in normal appearing tissue of the contralateral hemisphere has been described in patients with untreated glioblastoma, suggesting early tumor cell infiltration without disruption of the blood-brain-barrier [26].

BVZ has strong impact on tumor vessels, the blood-brain barrier and thus most likely on the osmotic environment. We therefore hypothesized that a metabolic reaction including a change of the MI concentration should be measurable during BVZ therapy. To investigate our hypothesis we evaluated the spectroscopic data of 30 prospectively evaluated patients with recurrent glioblastoma before and during therapy with BVZ. Another 9 patients with recurrent glioblastoma receiving CCNU (Lomustine) and VM26 (Teniposid) chemotherapy served as a control group. Compared to our previously published MRS study on the effects of BVZ, reporting data for the same patient cohort [8], we now present newly analyzed and unpublished data on a different metabolite as well as data on a newly recruited control group. Here we evaluated effects on the osmotic environment, while our previous study focused on phospholipid membrane metabolism and thereby on tumor cell proliferation.

## Methods

### Study design

This prospective, observational study was approved by the ethics committee of the Faculty of Medicine at the Johann Wolfgang Goethe-University Frankfurt (Reference number 4/09-SNO 01/09). Patients with a histological diagnosis of glioblastoma and radiologically confirmed recurrence based on the updated response assessment criteria (RANO) and a clinical recommendation for BVZ or CCNU/VM26 treatment were included. All patients gave their written informed consent. Patients received either BVZ (*BVZ cohort*) or CCNU and VM26 (*CCNU/VM26 cohort*. Patients of the *BVZ cohort* had been previously evaluated in a different MRS study [8] for which MI values had not been calculated. For unbiased results, we completely reanalyzed the existing data.

All participants underwent an MRS examination before the start of therapy and at regular MRI follow up. Follow ups were performed after 8 weeks for BVZ patients and 10–12 weeks for CCNU/VM26 patients.

In addition, we determined serum levels of VEGF-A before BVZ treatment using a commercially available ELISA kit (Quantikine®, R&D Systems, Minneapolis, MN) according to the manufacturer´s instructions.

### MR protocol and data analysis

MRS and MRI examinations were performed on a 3 Tesla whole body scanner (Magnetom Trio, Siemens Medical AG, Erlangen, Germany) using a double tuned ^1^H/^31^P volume head coil (Rapid Biomedical, Würzburg, Germany). The detailed MRS protocol can be found in the previously mentioned publication by Hattingen et al. [8]. The procedure and criteria for region of interest (ROI) and voxel selection which have also been published in said publication are recapitulated below for basic understanding.

A graphical user interface developed at our institute was used for the image guided selection of the tumor ROI based on T2-weighted and contrast enhanced T1-weighted images. A senior neuroradiologist (E.H., more than 10 years of experience) visually selected the voxels of the tumor area. This area was defined as solid contrast enhancing tumor mass without necrotic areas. We selected the control tissue from a contralateral region. In case of obvious tumor or edema in that region another adjacent unaffected area of the contralateral hemisphere was defined. During the follow-up examination under BVZ the tumor area frequently showed no or only faint contrast enhancement. The tumor area was then delineated based on signal changes in T2-weighted images according to following features [27]:

- intermediate signal intensity between grey matter and vasogenic edema,
- inhomogeneous signal changes,
- blurred gray-matter junction with altered cortical ribbon,
- lack of ‘fingers of edema’.

**Fig 1:**
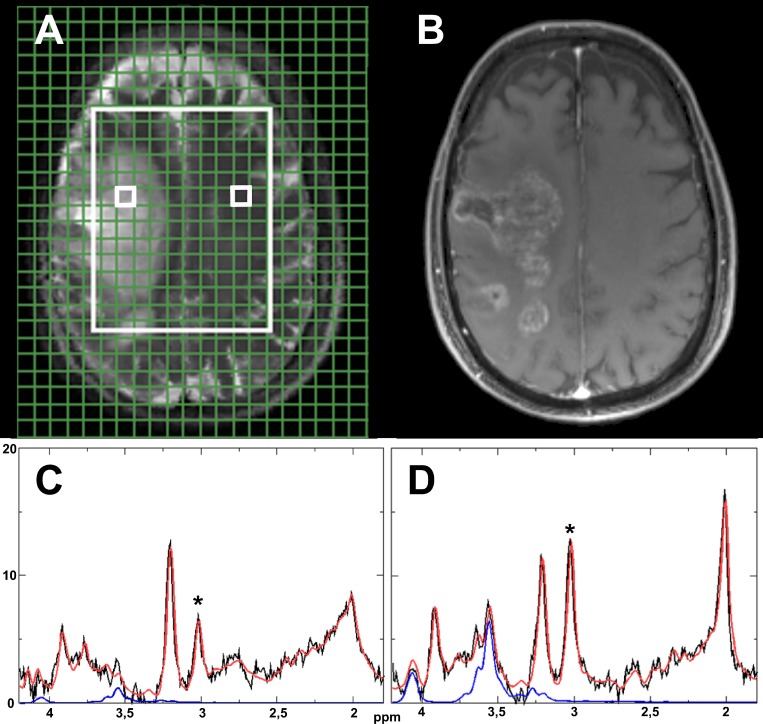
Model spectra. Graphical user interface on T2-weighted MR image with two small white boxes indicating an example voxel for tumor (left) and control tissue (right) within the measured area (large white box) (A). Corresponding T1-weighted contrast enhanced MR image (B).Corresponding spectra for the marked tumor (C) and control tissue voxels (D). The original signal is shown in black, the LCModel fit in red and the isolated signal for myoinsitol in blue. A * marks the creatine peaks.

The data was sampled from all feasible voxels within the tumor ROI and the control ROI. An additional ROI in between tumor and control tissue was not included due to the expected difficulty of attributing concentration changes to underlying mechanism with marked edema being visible in the morphologic images. We utilized the software LCModel (Provencher, downloadable test version at: http://s-provencher.com)[28] to calculate the MI concentration with the main resonances at 3.56 and 4.06 ppm, as described in detail by Hattingen et al. [8]. It should be mentioned that we did not use an absolute cutoff for the relative Cramér-Rao lower bounds (CRLB) calculated by LCModel, since the significance of the CRLB can be questionable for metabolites with a relatively low concentration like MI and a rigid cutoff might lead to an distortion of the sample distribution [29]. Nevertheless we report the CRLB values as orientation. As a robust quality marker we also analyzed the Cre concentration. We calculated the relative changes of the MI and Cre concentrations in the tumor and control tissue for every patient separately before calculating the average values. Fig 1 shows example images of one patient with the corresponding spectra.

### Statistical analysis

We performed statistical analysis with commercially available software (BiAS version 10.14, epsilon-Verlag; SPSS Statistics 22, IBM). To confirm the normal distribution of our data and the equality of variances we employed Shapiro–Wilk and F-tests.

We used a t-test to compare values between the BVZ and CCNU/VM26 cohort, between baseline and post treatment and between tumor and control tissue. Changes during treatment were calculated for each patient individually and then averaged. We compared the relative changes during treatment between the tumor and control tissue by Wilcoxon-matched-pairs test and we applied the Mann–Whitney U test to compare metabolites between independent groups. Cutoffs were calculated with a freely available, web based software (Cutoff Finder, http://molpath.charite.de/cutoff) [30].

### Survival analysis

Since most patients from the CCNU/VM26 cohort received BVZ at further progression the survival analysis was only done for the BVZ cohort. We split the group at the median overall survival time into a short overall survival subgroup (**short -OS**) and a long overall survival subgroup (**long -OS**). We then compared the MI values of these subgroups for tumor, control tissue and the difference between these tissues. Next we performed two Kaplan-Meier analyses, one for the relation between control tissue values at baseline and overall survival and one for the relation between baseline difference of tumor and control tissue (delta MI) and overall survival. In both cases we split the cohort at an optimized cutoff for differences in overall survival (generalized Wilcoxon test). P values < 0.05 were considered statistically significant for all analyses.

## Results

### Patient characteristics

Patient characteristics are shown in Table 1. A total of 39 patients were included. All patients were pretreated with radiochemotherapy with Temozolomide and adjuvant Temozolomide. Before being referred to BVZ therapy, patients usually received at least one cycle of a further chemotherapy (Lomustin).

**Table 1:** Patient characteristics.

Characteristics	*BVZ Cohort*	*CCNU/VM26 Cohort*
n	30	9
Sex		
• Female	8	3
• Male	22	6
Age		
• Median (range)	52 (31–69)	57 (48–66)
Histology		
• Primary GBM	26	8
• Secondary GBM	4	1
Previous therapies		
• No. of surgeries, median (range)	1 (0–4)	1 (0–3)
• No. of radiotherapies, median (range)	1 (1–4)	1 (1–2)
• No. of chemotherapies, median (range)	2 (1–6)	2 (1–2)
Recurrences		
• Median (range)	3 (1–5)	2 (1–3)
Concomitant therapy		
• Irinotecan	6	n/a
• Topotecan	1	n/a
• Radiotherapy	5	n/a

Patient characteristics, pretreatments and concomitant therapies for all patients in the *BVZ cohort* as well as the *CCNU/VM26 cohort*.

30 patients were treated with BVZ at a dose of 10 mg/kg body weight i.v. every other week. 6 of these patients additionally received irinotecan at a dose of 125 mg/m^2^, one patient received Topotecan at a dose of 0,4mg/ m^2^. Concomitant radiotherapy was performed in 5 patients. At baseline 21 patients were on dexamethasone and 20 patients received dexamethasone during the time of the follow up.

9 patients were treated with a combination of CCNU (90 mg/m^2^ i.v.) and VM26 (60 mg/m^2^ i.v.). Four patients were on dexamethasone at baseline and follow up. Five patients later switched to BVZ treatment. Hence, for the first time, we present data on both therapies in the same group of patients (*BVZ post CCNU* -subgroup).

### Results at baseline

Average CRLB values for the calculation of the MI concentration were 10.8% for the control tissue ROI and 22.2% for the tumor ROI. The respective CRLB values for the Cre concentration were 5.3% and 11.9%.

**Fig 2:**
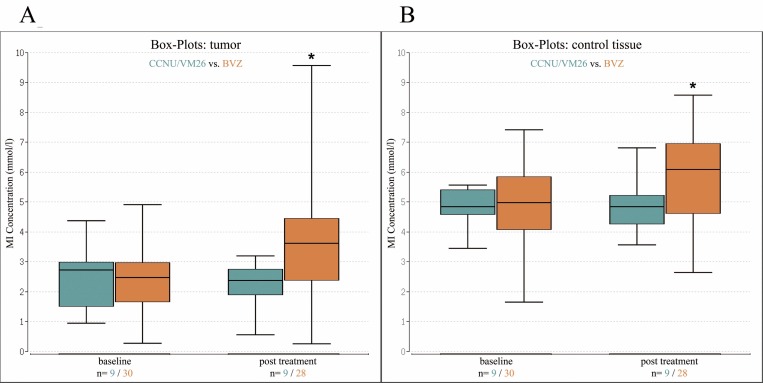
Box-Whisker-plots. (A) Box-Whisker-plot showing the median (baseline CCNU/VM26 = 2.73, BVZ = 2.47; post treatment CCNU/VM26 = 2.38, BVZ = 3.61) and quartiles for tumor at baseline and post treatment for BVZ (orange) and CCNU/VM26 (blue). (B) Box-Whisker-plot showing the median (baseline CCNU/VM26 = 4.83, BVZ = 4.97; post treatment CCNU/VM26 = 4.83, BVZ = 6.08) and quartiles for the control tissue at baseline and post treatment for BVZ (orange) and CCNU/VM26 (blue). Each * marks a significant increase from baseline to post treatment.

In both cohorts MI concentrations in the tumor were lower when compared to control tissue (p < .001 for both cohorts). MI concentrations for tumor and control tissue showed no significant difference between the BVZ and the CCNU/VM26 cohort at baseline (p = .48 for tumor, p = .39 for control) (Table 2, Fig 2A and 2B). The Cre concentrations in the tumor were also lower when compared to control tissue (p < .001 for both cohorts). None of the other comparisons including post treatment and survival data reached significance regarding Cre concentrations (Table 2).

**Table 2:** Results.

			baseline				post treatment				
	n		tumor	control	control-tumor	n	tumor	control	control-tumor	increase tumor (%)	increase control (%)
**BVZ**	30	MI	*2*.*42* (1.09)	*5*.*00* (1.49)		28	***3*.*66*** (2.00)	***5*.*85*** (1.51)		**93,8**	**32.0**
		Cre	4.03(1.67)	7.53 (1.65)			4.15 (1.72)	7.41 (1.39)		13.0	4.0
**CCNU/VM26**	9	MI	2.43 (1.07)	4.86 (0.65)		9	**2.24** (0.76)	**4.87** (0.91)		**3.7**	**1.2**
		Cre	4.19 (1.90)	6.56 (1.25)			4.41 (1.64)	6.59 (1.29)		13.6	2.7
-BVZ post CCNU	5	MI	*2*.32 (0.60)	4.76 (0.67)		*5*	*4*.*17* (1.29)	5.12 (0.76)		88.9	8.8
		Cre	5.10 (1.71)	5.94 (1.09)			5.18 (1.07)	6.67 (1.28)		13.0	14.9
**Long -OS**	15	MI	2.28 (0.66)	**5.65** (1.16)	**3.37** (1.28)	13	3.63(2.45)	6.39 (1.44)	2.76 (2.74)	63.6	17.1
		Cre	4.33 (1.79)	7.68 (0.95)	3.35 (1.87)		3.91 (2.16)	7.74 (1.44)	3.83 (1.67)	-5.5	4.7
**Short -OS**	15	MI	2.55 (1.41)	**4.36** (1.54)	**1.81** (2.04)	15	3.69 (1.60)	5.38 (1.45)	1.69 (2.17)	120.1	45.0
		Cre	3.74 (1.55)	7.38 (2.17)	3.63 (2.60)		4.36 (1.28)	7.12 (1.33)	2.77 (1.88)	29.0	3.4

Mean values and standard deviations (in brackets) for the MI and Cre concentration (mmol/l) in the tumor, contralateral control tissue (control), control tissue minus tumor and the relative increase during treatment. **Boldface** font indicates a significant difference between either the BVZ and CCNU/VM26 cohorts or the long -OS and short -OS. *Italic* font indicates a significant difference between baseline and post treatment. Note that baseline and post treatment values for the BVZ cohort are calculated based on a different number of patients (n = 30 vs. n = 28) since fully evaluable follow up data was not available in 2 patients. Thus the same applies for the Long -OS and Short–OS subgroup (n = 13 vs. n = 15).

### Results post treatment

Fully evaluable baseline and follow-up MRS was available in 28 of 30 patients receiving BVZ, and in all patients in the CCNU/VM26 cohort. In the CCNU/VM26 group, MI levels did not change post treatment (Table 2, Fig 2A and 2B). In the BVZ group, MI values increased significantly post treatment compared to baseline, for both tumor and control tissue (p < .001 for tumor, p = .002 for control). The relative changes were more pronounced in the tumor compared to control tissue (tumor 93.8% increase, control 32.0% increase, p = .049). Even though the absolute difference between tumor and control tissue decreased, tumor values remained significantly lower (p < .001).

Absolute MI levels for tumor and control tissue of the BVZ cohort were significantly higher post treatment compared to corresponding values in the CCNU/VM26 cohort (p = .023 for tumor, p = .038 for control) (Table 2, Fig 2A and 2B).

The five CCNU/VM26 patients who received BVZ at further progression represent an opportunity to compare effects of both treatments in the same patient. In contrast to the CCNU/VM26 treatment the MI levels during BVZ therapy did increase markedly in the tumor (p = .04). A smaller increase of MI in the control tissue was noted as well (Table 2).

### Survival analysis

**Fig 3:**
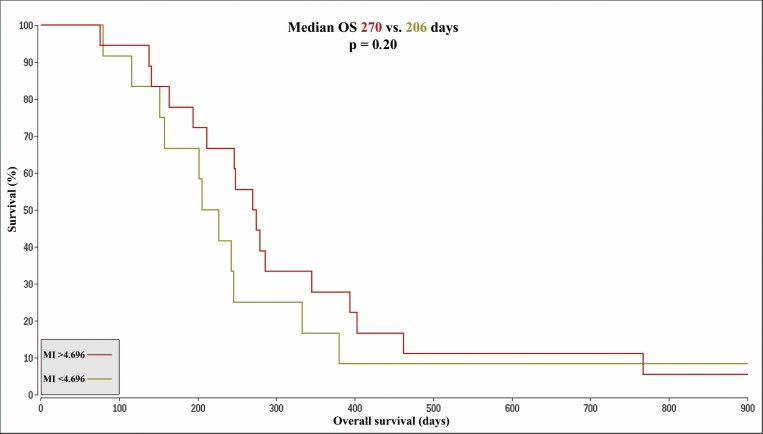
Kaplan-Meier-Curves based on control tissue MI. Kaplan-Meier-Curves for patients with a MI concentration in the control tissue at baseline > 4.696 (OS 270 days, color: red) compared to patients with a MI concentration < 4.696 (median overall survival (OS 206 days, color: brown).The figure shows an extract with an OS of 1829 days for the remaining patient of the brown cohort and a censored survival of 1424 days for the remaining patient in the red cohort. MI = Myoinositol; OS = overall survival; delta MI = MI control tissue minus tumor concentrations at baseline.

**Fig 4:**
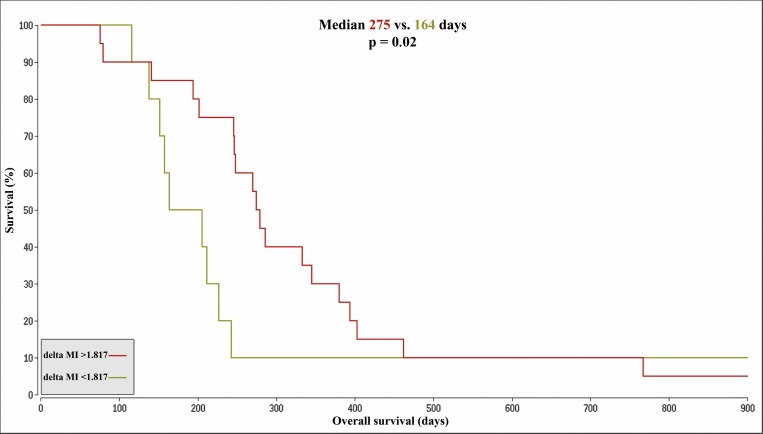
Kaplan-Meier-Curves based on delta MI. Kaplan-Meier-Curves for patients with delta MI values > 1.817 (OS 275 days, color: red) compared to patients with values < 1.817 (OS 164 days, color: brown). The figure shows an extract with an OS of 1829 days for the remaining patient of the brown cohort and a censored survival of 1424 days for the one remaining patient in the red cohort. MI = Myoinositol; OS = overall survival; delta MI = MI control tissue minus tumor concentrations at baseline.

The median survival in the BVZ cohort (n = 30) was 246.5 days. The respective 15 patients with a longer survival (long–OS) had a median survival of 345 days. The 15 patients with a shorter overall survival (short -OS) had a median survival of 164 days. The MI levels in the tumor did not differ significantly between the two subgroups. In contrast, MI levels in the control tissue at baseline and at follow up were significantly elevated in the long -OS group (p = .008, p = .039) (Table 2). An optimized cutoff was set at a MI concentration of 4.696 and a Kaplan-Meier analysis for the values of the control tissue at baseline was performed as shown in Fig 3. Median survival for the high MI subgroup (n = 18) was 270 days in contrast to 206 days for the low MI group (n = 12, p = .20). The difference between the tumor and control tissue MI concentration at baseline (delta MI) was significantly higher in the long -OS than in the short -OS group (p = .009). A Kaplan-Meier analysis with an optimized cutoff of 1.817 for delta MI is shown in Fig 4. The median survival difference between the subgroup with a higher delta MI (n = 20, median OS 275 days) and the subgroup with a lower delta MI (n = 10, median OS 164 days) was 111 days and reached significance (p = .022).

For the relative increase of the MI concentrations in the tumor or the control tissue a relation to overall survival could not be demonstrated (S1 and S2 Figs).

### VEGF serum levels

We were able to measure serum VEGF-A serum levels in 28 out of 30 patients within the BVZ cohort before the start of BVZ therapy. The VEGF-A levels in the long–OS subgroup did not differentiate from the levels in the short–OS subgroup (261 pg/ml for long–OS, 358 pg/ml for short–OS, p = .227). The same applies for the patients with different delta MI (Table in S1 Table).

## Discussion

In this prospective MRS study in patients with recurrent glioblastoma treated with BVZ we were able to show that the MI concentration in tumor tissue and contralateral control tissue increases significantly with BVZ therapy. Moreover, higher baseline MI levels in unaffected control tissue and a higher difference to the concentration in tumor tissue (delta MI) were significantly associated with longer overall survival.

MI is an isomer of glucose which is acting as an organic osmolyte in the brain [31]. Its concentration in the cells is regulated depending on the environmental osmolality, regardless of a certain kind of osmotic disorder [13–15,17]. Therefore, the MI concentration may indicate a metabolic reaction to osmotic changes in the brain. A change of the total MI concentration triggered by changes in membrane or second messenger metabolism seems less likely, due to the variety of metabolic pathways facilitating MI [12] and has to our knowledge not been described.

The distinct reduction of contrast enhancement and peritumoral edema during BVZ therapy [32] suggests an impact of BVZ on the osmotic environment in glioblastoma. The VEGF secretion of glioblastoma causes a disruption of the blood-brain barrier followed by a leakage of electrolytes and small molecules from the vessels which leads to massive, vasogenic edema [33]. BVZ blocks this VEGF pathway and its strong in vivo impact on tumor vessels has been described by several MRI studies using different methods [34,35]. Therefore, it seems reasonable to assume a distinct shift in the osmotic environment during therapy. In this context, our findings are consistent with studies on central pontine myelinolysis which reported almost doubled MI concentrations for control subjects or after correction of the underlying hyponatremia [13–15]. Thus, the measured increase of the MI concentration of about 94% in the tumor due to BVZ treatment was rather expected. In contrast, we did not observe changes of the MI concentrations during cytotoxic CCNU/VM26 therapy. Consequently the post treatment MI values in the tumor tissue were significantly higher in the BVZ cohort compared to the CCNU/VM26 cohort. The subgroup from the CCNU/VM26 cohort who received BVZ at further progression, again showed an increase of MI comparable to the BVZ cohort. This independently supports our observations from the BVZ cohort. We could furthermore exclude that systematic differences at baseline between the CCNU/VM26 and the BVZ cohorts caused different MI reactions upon treatment (Tables 1 and 2). A purely physical explanations for increasing MI levels, like an increased cell density [26] can be mostly excluded since Cre concentrations remained unchanged. The low MI and Cre concentration in the tumor compared to the control tissue are in accordance with previous MRS studies [21–25,36]. Since other metabolites like Choline and N-acetylaspartate did also not show significant changes or relations to overall survival in a previous study [8], we will not further discuss these metabolites in this publication (further metabolite data for the CCNU/VM26 cohort can be found in S2 Table).

Under BVZ we did not only observe a relative increase of the MI concentration in the tumor, but also in the normal appearing control tissue of the contralateral hemisphere. MI increased about 32% in the control tissue under BVZ treatment and post treatment values were significantly higher in the BVZ cohort than in the CCNU/VM26 cohort. The change of the MI concentration in the control tissue could suggest an impact of BVZ on healthy tissue or an increase through astrocytic gliosis. Since no changes in other metabolites were notable [8], the control tissue remained unaffected on standard T2 sequences and the timespan to the second MRSI examination was rather short, we do not consider astrocytic gliosis as a likely explanation. Alternatively a tumor related decrease of MI could already be present at baseline, with BVZ then exerting an effect comparable to the one in tumor tissue. Compared to our measurements studies on healthy adults reported both higher and lower baseline MI concentrations [37,38]. This indicates that methodical and technical differences impede the comparison of absolute values between different studies. To further investigate this hypothesis of a tumor related effect we performed survival analyses.

The dichotomization of the BVZ cohort into a group with long -OS and one with short -OS at the median survival time revealed significantly higher MI concentrations in the control tissue at baseline for long -OS patients. The concentrations of MI in the tumor did not differ significantly between these two groups. Consistently, the delta MI was significantly larger in the long -OS group. With an optimized cutoff, especially the delta MI values were predictive of overall survival (Fig 4), meaning lower MI concentrations in the control tissue and a smaller delta MI where associated with shorter OS. Altogether these observations could point to an affection of the contralateral control tissue at baseline.

We assume that this could either be explained by direct tumor cell infiltration or an indirect tumor effect on the contralateral hemisphere. These indirect effects might include spreading edema that is not yet visible in MRI scans, transfer of cytokines like VEGF to the contralateral hemisphere or just increased intracranial pressure affecting the contralateral hemisphere. Through our measurements we can fairly exclude the possibility of elevated VEGF serum levels affecting contralateral healthy brain tissue. The diffusion of cytokines or the spreading of a mild, invisible edema over such a long distance and through the fornix seems rather unlikely and has to our knowledge not been described in this context. In contrast, a high grade glioma invasion to the contralateral hemisphere has already been proven to occur in more than 50% of untreated tumors by Matsukado et al. in 1961 [39] and since has been further validated [40]. We therefore assume tumor cell invasion and its influence on the osmotic environment to be the best hypothesis for the deviation and probably decreased MI concentration in the contralateral control tissue at baseline. A similar idea, employing MI as tumor invasion marker, has been described by Kallenberg et al. in 2009 [37]. Their spectroscopic examination of 22 newly diagnosed glioblastoma patients and 14 age matched control subjects yielded altered concentrations of MI in the contralateral hemisphere of the glioblastoma patients compared to the control subjects. Following this reasoning, the MI concentration in the control tissue could be a marker for the extent of tumor infiltration that is more sensitive than morphologic MRI scans.

We have to mention that the additional medication with dexamethasone might cause at least a small change in the MI concentration [41,42]. However a MRI study on GBM patients did not show any effect of dexamethasone on cerebral blood flow, cerebral blood volume, mean transit time and water mean diffusivity in normal appearing brain tissue and only a small change of cerebral blood flow and water mean diffusivity in the tumor [41]. In concordance a MRS study on GBM patients was not able to assess different MI levels in the control tissue depending on dexamethasone treatment [43]. Compared to BVZ, dexamethasone was significantly less effective in decreasing the blood volume and permeability in the tumor in a MRI study on a glioblastoma rat model [35]. In summary the effect of dexamethasone, if present, might be too small to be detected in our patient cohort.

### Limitations

A general limitation certainly is the small number of patients we examined. Especially for the survival analysis, a larger collective would be needed to explore whether MI can be a clinically useful predictive marker. Another restriction is that we cannot completely distinguish between MI and Glycine with the technical methods applied. Since the proportion of Glycine in the signal is believed to be less than 10% [19] and Glycine levels in opposition to MI levels are known to be elevated in glioblastoma [21,23,24,44,45] we assume that the Glycine fraction did not contribute to a relevant extent. Finally, partial volume effects in the tumor measurements can never be fully excluded due to the strong inhomogeneity with compact tissue next to necrosis and edema. But our measurements in the control tissue are far less susceptible for this pitfall because metabolite concentrations in homogenous tissue are very stable within adjacent voxels and thus more independent from the ROI.

### Conclusion

Comparing BVZ to a cytotoxic treatment we confirmed that glioblastoma shows a strong metabolic reaction to BVZ. Furthermore, we identified pre-therapeutic MI levels to be a predictive marker of OS under BVZ therapy. Our results suggest that MI may be a more sensible marker for tumor cell invasion than regular MR imaging.

## Supporting Information

S1 Table VEGF serum levels. (XLSX)

S2 Table Additional metabolite concentrations for the CCNU/VM26 cohort. Mean values and standard deviations (in brackets) for metabolite concentrations (mmol/l) in the tumor and contralateral control tissue (control).
(XLSX)

S1 Fig Kaplan-Meier-Curves based on the MI change during treatment in tumor. An optimized cutoff was set at a MI change of 1.283.The figure shows an extract with an OS of 1829 days for the remaining patient of the brown cohort and a censored survival of 1424 days for the remaining patient in the red cohort. MI = Myoinositol; OS = overall survival; MI change = MI increase in tumor during treatment.
(TIF)

S2 Fig Kaplan-Meier-Curves based on the MI change during treatment in control tissue. An optimized cutoff was set at a MI change of 1.445.The figure shows an extract with an OS of 1829 days for the remaining patient of the brown cohort and a censored survival of 1424 days for the remaining patient in the red cohort. MI = Myoinositol; OS = overall survival; MI change = MI increase in control tissue during treatment.
(TIF)
