# Supplementary figures and images for: Myoinositol as a Biomarker in Recurrent Glioblastoma Treated with Bevacizumab: A 1H-Magnetic Resonance Spectroscopy Study
Source: PLoS One. 2016 Dec 29;11(12):e0168113. doi: 10.1371/journal.pone.0168113 (PMC5198997; doi:10.1371/journal.pone.0168113)

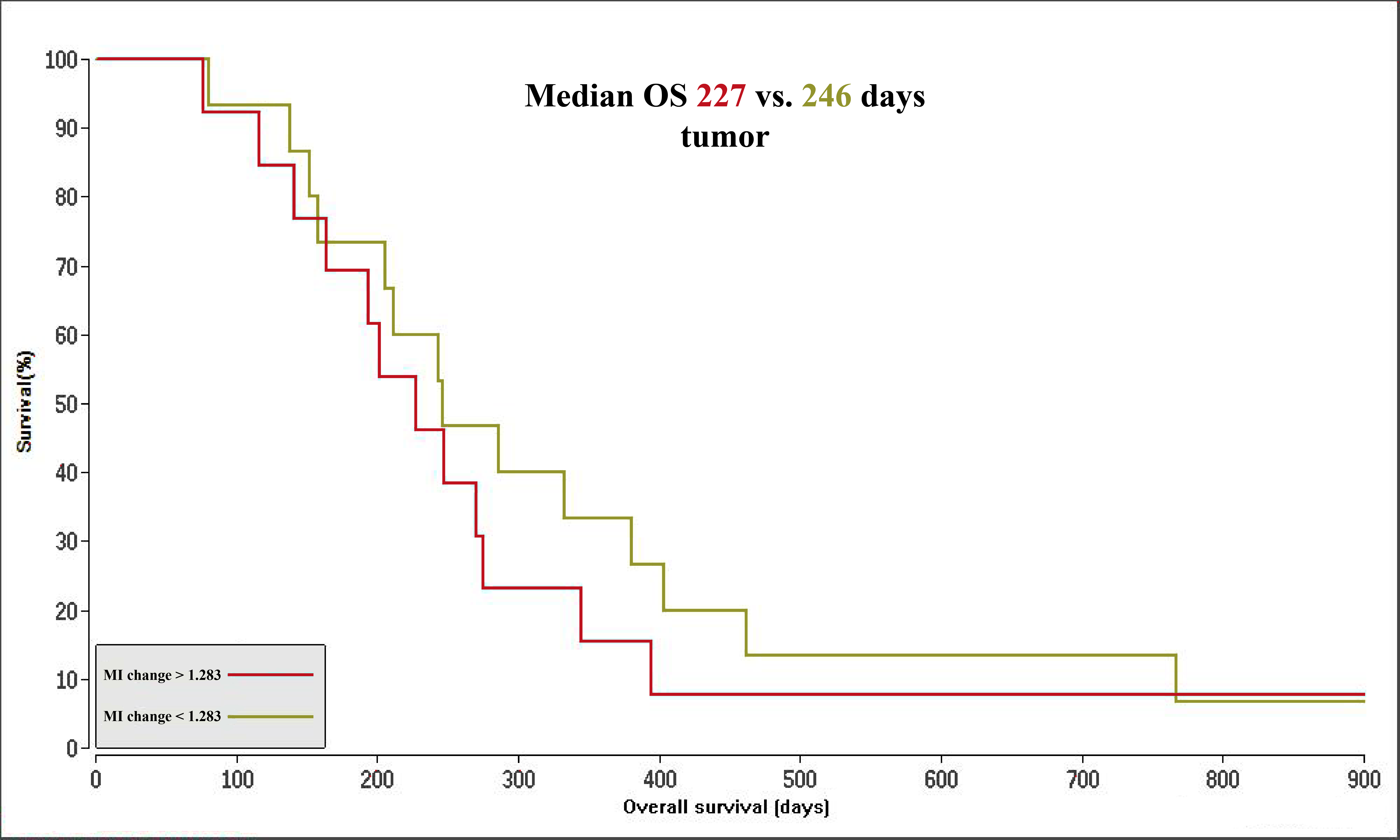

Supplement: S1 Fig — An optimized cutoff was set at a MI change of 1.283.The figure shows an extract with an OS of 1829 days for the remaining patient of the brown cohort and a censored survival of 1424 days for the remaining patient in the red cohort. MI = Myoinositol; OS = overall survival; MI change = MI increase in tumor during treatment. (TIF) [file pone.0168113.s003.tif]

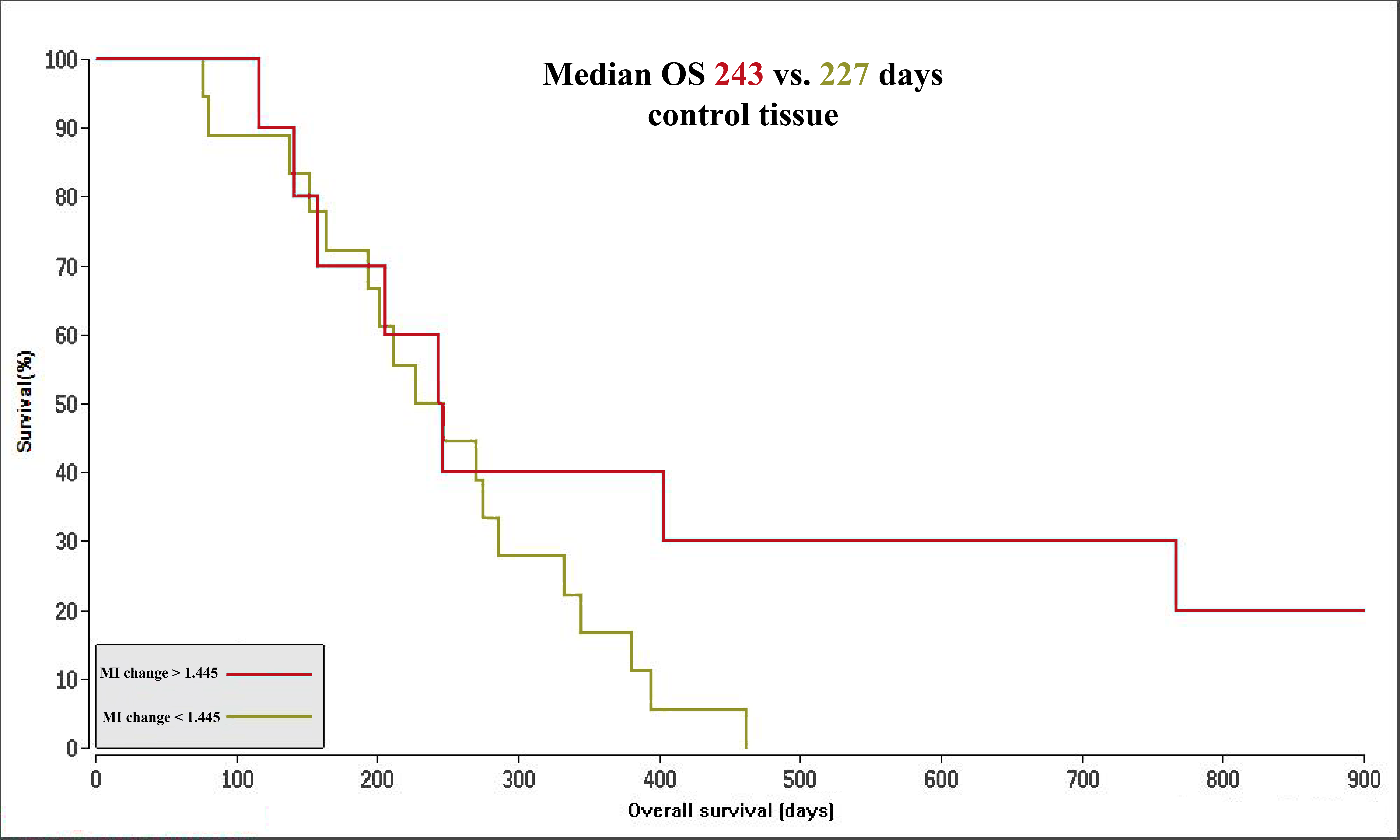

Supplement: S2 Fig — An optimized cutoff was set at a MI change of 1.445.The figure shows an extract with an OS of 1829 days for the remaining patient of the brown cohort and a censored survival of 1424 days for the remaining patient in the red cohort. MI = Myoinositol; OS = overall survival; MI change = MI increase in control tissue during treatment. (TIF) [file pone.0168113.s004.tif]
